# The true cost of red cell transfusion for patients with myelodysplastic syndromes: A time‐driven activity‐based costing study

**DOI:** 10.1111/bjh.70556

**Published:** 2026-05-21

**Authors:** Allison Mo, Helen Haysom, Alayna Carrandi, Sara Carrillo de Albornoz, Jessica Guglielmino, Kylie Rushford, Amanda Ellison, Dan Andrew, Angelene Jesurajah, Erin Hu, Philomina Banahene, Loo Sin Hoo, Alisa M. Higgins, Jake Shortt, Erica M. Wood, Zoe K. McQuilten

**Affiliations:** ^1^ Transfusion Research Unit, Faculty of Medicine, Nursing & Health Sciences School of Public Health & Preventive Medicine, Monash University Melbourne Australia; ^2^ Monash Haematology Monash Health Melbourne Australia; ^3^ Austin Pathology and Department of Haematology Austin Health Melbourne Australia; ^4^ Australian and New Zealand Intensive Care Research Centre Monash University Melbourne Australia; ^5^ Centre for Health Economics Monash University Melbourne Australia; ^6^ Monash Pathology Monash Health Melbourne Australia; ^7^ Pharmacy Department Monash Health Melbourne Australia; ^8^ Medical Infusion Unit Monash Health Melbourne Australia; ^9^ Department of Medicine, Faculty of Medicine, Nursing & Health Sciences School of Clinical Sciences, Monash University Melbourne Australia; ^10^ Department of Haematology Alfred Health Melbourne Australia

**Keywords:** costing, myelodysplastic syndromes, transfusion

## Abstract

Patients with myelodysplastic syndromes (MDS) often require red blood cell (RBC) transfusion. However, the true cost of RBC transfusion, beyond the acquisition cost of the unit, is poorly defined. We conducted a prospective time‐driven activity‐based costing study at a large Australian university teaching hospital. Every activity relating to RBC transfusion in patients with MDS was observed and process‐mapped, ranging from patient pre‐transfusion phlebotomy through to laboratory processes, clinical transfusion in inpatient and outpatient wards, clinical follow‐up and transfusion governance and oversight. For each activity, detailed data collection was undertaken, including timing of individual steps and recording all consumables, equipment and personnel involved. The total per‐unit cost was US$440.47, with an annual per‐patient estimated cost of US$6607.12. Hospital‐related costs contributed 41% of the total cost, with the largest contributor being equipment (16%) followed by staffing (14%). Alloimmunisation increased process costs by 24%. These findings demonstrate that hospital process costs need to be included when considering and budgeting for the total cost of the transfusion process. Our findings will inform future transfusion‐related resourcing and cost‐effectiveness evaluations of treatments which aim to reduce RBC transfusion burden and improve quality of life in MDS.

## INTRODUCTION

Red blood cell (RBC) transfusions are a cornerstone of supportive care in patients with myelodysplastic syndromes (MDS), yet their true cost goes far beyond the price of the blood unit. The cost charged for one RBC unit varies internationally^1^, ^2^; however, this cost typically only covers procurement costs related to blood collection and processing^1^ and not additional costs related to transfusion, such as phlebotomy, laboratory processes, ward transfusion processes, clinical monitoring following transfusion and many other essential administrative and hospital processes.

Understanding the true cost enables government and health services to allocate future funding and resources appropriately and evaluate the cost‐effectiveness of emerging therapies, such as luspatercept, an erythroid maturation agent or imeltestat, a telomerase inhibitor, which can reduce RBC transfusion requirements^3^ but may be costly.

Anaemia is nearly universal in patients with MDSs. Approximately one‐third of patients with MDS are transfusion dependent^4^ making them among the highest users of RBC transfusion.^5^, ^6^ Previous transfusion costing studies have focused on other patient populations, such as thalassaemia,^7^ surgery^8^ or more broadly within all hospitalised patients,^9^ which may not reflect the needs of patients with MDS, who are typically older, often have comorbidities and higher rates of alloimmunisation.^10^

In this study, we aimed to determine the true total cost of providing RBC transfusion support to patients with MDS by measuring every step of the transfusion pathway in a real‐world setting, using a time‐driven activity‐based costing (TDABC) model. The TDABC model accounts for all staff, equipment, consumables, the time taken for each activity and the probability/frequency of different activities occurring, to calculate the overall cost of transfusion.^11^, ^12^, ^13^, ^14^

## METHODS

### Study design

This was an observational prospective study using TDABC model from a healthcare provider perspective to estimate the total cost of the transfusion process. Unlike traditional activity‐based costing (ABC) models, which allocate costs using predefined activity drivers, TDABC assigns costs based on the actual time and capacity used for each process step, providing greater accuracy and transparency.^15^ TDABC uses a bottom‐up micro‐costing approach to generate precise cost information for each unit of activity and measure costs across the continuum of care.^16^ It has been proposed as an improvement to traditional ABC costing because it makes ABC faster and easier to update by transforming all cost drivers into a single cost driver (time) and avoids reliance on broad activity allocations.^15^

In line with published standardised TDABC methodology frameworks,^11^ we observed and process‐mapped every activity related to the transfusion episode including all staff, equipment, consumables, the time taken for each activity and the probability/frequency of different activities occurring. These were then combined to calculate the overall cost of the transfusion process.^11^, ^12^, ^13^

This study was approved by the Monash Health Research Ethics Committee (reference number RES‐22‐0000‐007Q‐81488).

### Study population and setting

This study was conducted at Monash Medical Centre (MMC), a 640‐bed tertiary‐level university hospital in Melbourne, Australia. From May to October 2022, we observed patients with MDS receiving RBC transfusion in the inpatient haematology ward and two outpatient transfusion day wards.

### Data collection

The RBC transfusion episode was divided into four phases:

1. Pre‐transfusion phlebotomy
2. Laboratory processes:
  - specimen reception
  - transfusion laboratory processes
  - cross‐match and laboratory issue of blood product
3. Clinical transfusion processes
4. Additional processes relating to transfusion

Data collection sheets describing individual processes within each phase were developed with input from haematologists, laboratory and nursing staff, then piloted and refined. Each process was mapped into flowcharts, including ‘decision diamond’ points for alternate pathways. Each activity was observed and timed at three times using a convenience sample, with averages calculated; additional observations were made for highly variable tasks. Infrequent events (e.g. severe transfusion reactions) which were not directly observed were estimated by expert opinion. All data were checked for accuracy and consistency by staff from the relevant departments.

### Apportioning costs and resources

The 2023 procurement cost price for one RBC unit—universally leucodepleted, either irradiated or non‐irradiated—from volunteer donors, supplied by Australian Red Cross Lifeblood, was US$258.27 (AU$357.14)^1^ covering collection, processing and distribution.

Unit costs of individual consumables, equipment and staff wages were obtained from hospital Human Resources, Finance, Procurement departments and published enterprise agreements.^17^, ^18^, ^19^ Staff wages included on‐costs of 30% of the base salary to account for additional costs of compulsory national superannuation, workers' compensation and leave entitlement.^20^ For processes involving staff of different seniority levels, a weighted average was estimated based on rosters or expert opinion.

The cost of equipment per RBC transfusion episode was calculated using equipment acquisition costs, maintenance costs and estimated life‐years. We did not include non‐clinical equipment and consumables (e.g. computers, printers) as these are used widely across multiple hospital functions, and their contribution to the transfusion process for a patient with MDS is negligible. Similarly, hospital infrastructure costs were excluded. Given that hospital buildings are used over extended periods (many decades) and serve a wide range of patients and activities, their contribution to the cost of transfusing a single unit of RBC to an MDS patient is likely minimal. In addition, accurately attributing or proportioning these shared costs is methodologically challenging due to their broad and overlapping use and therefore outside the scope of the analysis.

Using TDABC methodology, hospital activity data over the 6‐month observation period were collected to inform cost calculations.^15^ For example, the percentage of time spent on MDS patient RBC transfusion‐related work versus other patient work informed apportioning of staff wage and equipment cost. Data also informed the probabilities at the ‘decision diamonds’ (e.g. proportion of patients with alloantibodies informed probability of additional antibody testing occurring). The following representative data were collected:

- Hospital records for all patients with MDS receiving RBC transfusion (May–October 2022), including number of units transfused, inpatient and outpatient transfusion admissions, transfusion reactions and management, use of transfusion‐related medications (e.g. iron chelation, pre‐medications, diuretics).
- Hospital admissions, including total inpatient and outpatient admissions and proportion of these involving MDS patients receiving RBC transfusion.
- Laboratory data, including laboratory testing volumes (e.g. blood group & antibody screens), alloimmunisation rates, use of special blood products (e.g. extended phenotype‐matched RBCs), proportion of total RBC units issued to MDS patients.

This study was conducted from the healthcare provider perspective, and therefore, only includes activities and costs borne by the healthcare system^21^; thus, out‐of‐pocket costs to patients (e.g. transport) or societal costs (e.g. productivity loss) were not included.

As data analysis commenced in 2023, costs were based on 2023 values, calculated in Australian dollars and converted to US dollars using Organisation for Economic Co‐operation and Development Power Purchasing Parity rates.^22^

### Process maps, data analysis and cost calculations

**FIGURE 1:**
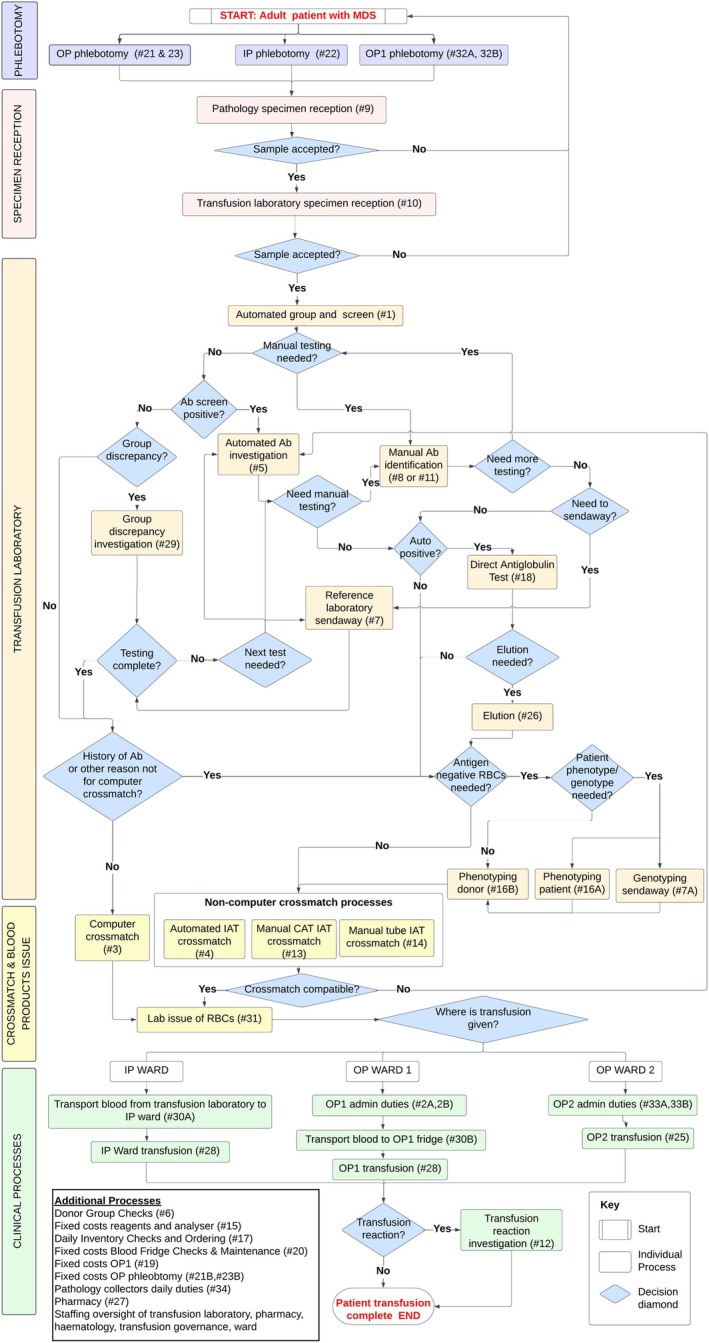
Master flowchart of all processes. Master flowchart showing links between individual process maps as numbered, and decision diamonds. Processes are divided into phlebotomy, specimen reception, transfusion laboratory, cross‐match & blood product issue and clinical processes. Ab, antibody; ARCLB, Australian Red Cross LifeBlood; CAT, column agglutination technology; IAT, indirect anti‐globulin test; IP, inpatient ward; OP1, outpatient ward 1; OP2, outpatient ward 2; RBC red blood cell.

In total, 34 process maps were developed detailing over 600 individual activities (four phlebotomy, 22 laboratory, including pathology specimen reception, blood bank investigations, cross‐match and laboratory issue, and eight clinical) and combined into a master flowchart (Figure 1). Figure S1 depicts example process maps from each of the different phases of the transfusion process. The probabilities of different decision diamond pathways in the process maps are detailed in Table S1.

**FIGURE 2:**
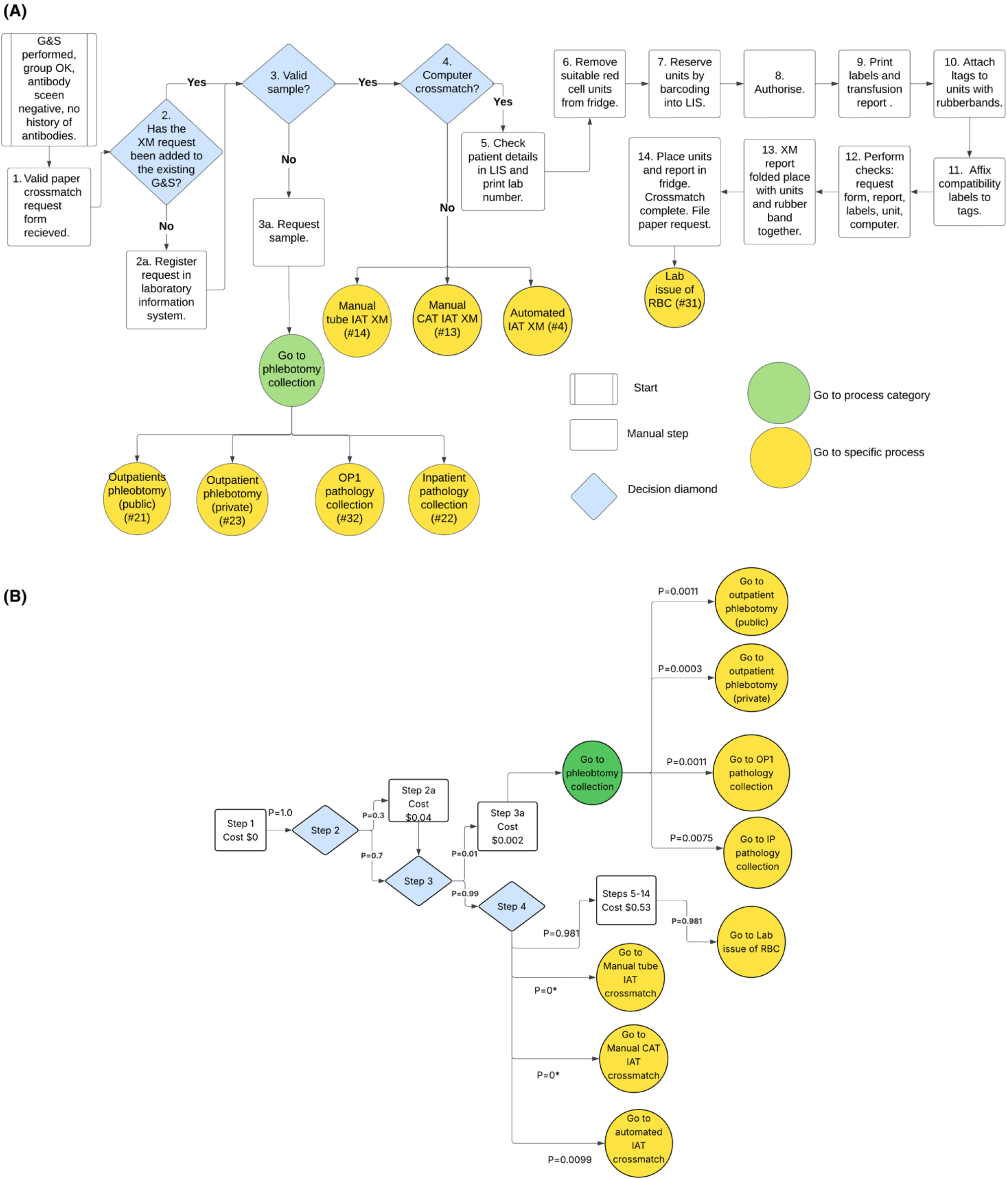
(A) Example of an individual process flowchart: Computer cross‐match with paper request form. Manual steps are shown in white rectangular boxes, decision diamonds are in blue. The probabilities at each blue decision diamond are determined by representative data from the study period or from expert opinion. This flowchart then leads onto other processes (yellow and green circles). CAT, column agglutination technology; IAT, indirect anti‐globulin test; LIS, laboratory information system; OP1, outpatient ward 1; RBC, red blood cell; XM, cross‐match. (B) Calculation of the weighted cost of computer cross‐match with paper request form. Flowchart from Figure 2A with cost and probabilities (denoted by ‘*p*=’) at each step indicated. Weighted cost is obtained by multiplying probability by cost at each step. *Note probabilities may be 0 for rare events, as confirmed by the representative data. CAT, column agglutination technology; IAT, indirect anti‐globulin test; IP, inpatient; LIS, laboratory information system; OP1, outpatient ward 1; RBC, red blood cell; XM, cross‐match.

The base‐case model of per‐unit cost of RBC transfusion was calculated by:

1. All components (consumables, staff time, equipment) in each process map step were identified and costed.
2. Weighted costs for each process map were calculated by multiplying step costs by their probabilities and summing these, as illustrated in Figure 2. Table S2 lists the weighted cost of each process map.
3. Process maps were classified as follows:
  - variable processes—differing between different transfusion episodes (e.g. inpatient vs. outpatient transfusion); or
  - fixed processes—routine per‐unit costs (e.g. daily laboratory quality control activities).
4. Total variable process costs were obtained by summing the weighted costs of variable process maps multiplied by their probabilities within the master map (Figure 1). Fixed costs were apportioned per RBC unit transfused and added to the variable costs.

Sensitivity analyses were conducted to explore the impacts of changes in timing and probability estimates. Although emphasised as an important consideration in TDABC studies, there are varying approaches which may be used.^16^, ^23^ There is no universally accepted rule for the magnitude of variation when performing sensitivity analyses in TDABC studies, and prior TDABC studies have varied timings and probabilities using different ranges, depending on the context and study objectives.^24^, ^25^ For this study, we selected a variation of ±10% of all recorded timings and ±10% variation on the probabilities of individual steps occurring, reflecting a pragmatic and plausible variation on these parameters in this particular study setting, as informed by expert opinion, similar to previous studies.^25^

Costs were also compared for patients with and without alloimmunisation.

Analyses were conducted using R version 4.4.2.^26^

## RESULTS

### Hospital activity data and process maps used in cost allocation and calculations

From May to October 2022, 26 patients with MDS were transfused 290 RBC units in MMC, including 92 inpatient ward transfusion episodes and 77 outpatient ward transfusion episodes, with a median of 1 (interquartile range [IQR] 1–2) RBC units per episode. More detailed transfusion‐related data used in the cost analysis are shown in Tables 1 and 2. Individual consumables, equipment and staffing resources included in the cost calculations are detailed in Table S3.

**TABLE 1:** Transfusion‐related data from May to October 2022 used in cost analysis.

	Number
Hospital RBC product and transfusion data	
Total RBC units received	8934
Total RBC units discarded	90
Total RBC units transfused	8844
Total RBC units transfused to MDS patients^a^ (%)	290 (3.3%)
Total no. MDS patients transfused across all wards	26
Total no. MDS RBC transfusion episodes	203
No. of transfusion episodes per transfused MDS patient, median (IQR)	5.5 (1–9)
No. RBC units transfused per MDS patient, median (IQR)	7.5 (3–13)
No. RBC units transfused per MDS transfusion episode, median (IQR)	1 (1–2)
Transfusion‐related testing	
Total *N* of blood group and antibody screen (G&S) samples processed	13 086
*N* of G&S samples from MDS patients processed (% of total G&S samples)	248 (1.9%)
Total *N* of MDS patient samples processed for computer cross‐match	148
Total *N* of MDS patient samples processed for IAT cross‐match	14
No. of MDS patients with alloantibodies	7
No. of extended‐phenotype matched RBC units requested	12

Abbreviations: IAT, indirect anti‐globulin test; MDS, myelodysplastic syndromes; RBCs, red blood cells.

^a^ This also includes RBC units transfused to patients in locations other than the wards included in this study (33 RBC units in trials department, 19 units in emergency department). These departments were not included in the costing study as the processes and costs are specific to the department and are likely to depend on other factors such as trial protocol or possibility of massive bleeding or trauma contributing to transfusion needs in the emergency department.

**TABLE 2:** RBC transfusion characteristics of inpatient and outpatient wards during May–October 2022.

Characteristics	Inpatient ward	Outpatient ward 1	Outpatient ward 2
Total no. patient admission episodes	730	5336	1762
Total no. MDS patient transfusion admission episodes	92 transfusion episodes in 16 inpatient admissions	68	9
Total number of RBC units transfused to all patients	766	705	67
Total number of RBC units transfused to MDS patients	106	118	14
Total no. MDS patients transfused	11	14	4
Number of transfusion episodes per patient, median (IQR)	4 (1–5)	4.5 (1–8)	2.5 (1.5–3)
Number of RBC units received per patient over time period, median (IQR)	4 (1–7)	6 (3–13)	3 (2–5)
RBC units per transfusion episode, median (IQR)	1 (1–1)	2 (1–2)	2 (1–2)
No. of adverse transfusion reactions documented	4	0	0

Abbreviations: MDS, myelodysplastic syndromes; RBCs, red blood cells.

### Total cost of transfusion

After incorporating the weighted costs of each process within the TDABC model, these were then apportioned according to the estimated frequency of each individual process occurring in the transfusion of an individual RBC unit to a patient with MDS. The total estimated cost per RBC unit transfused was calculated to be US$440.47.

**FIGURE 3:**
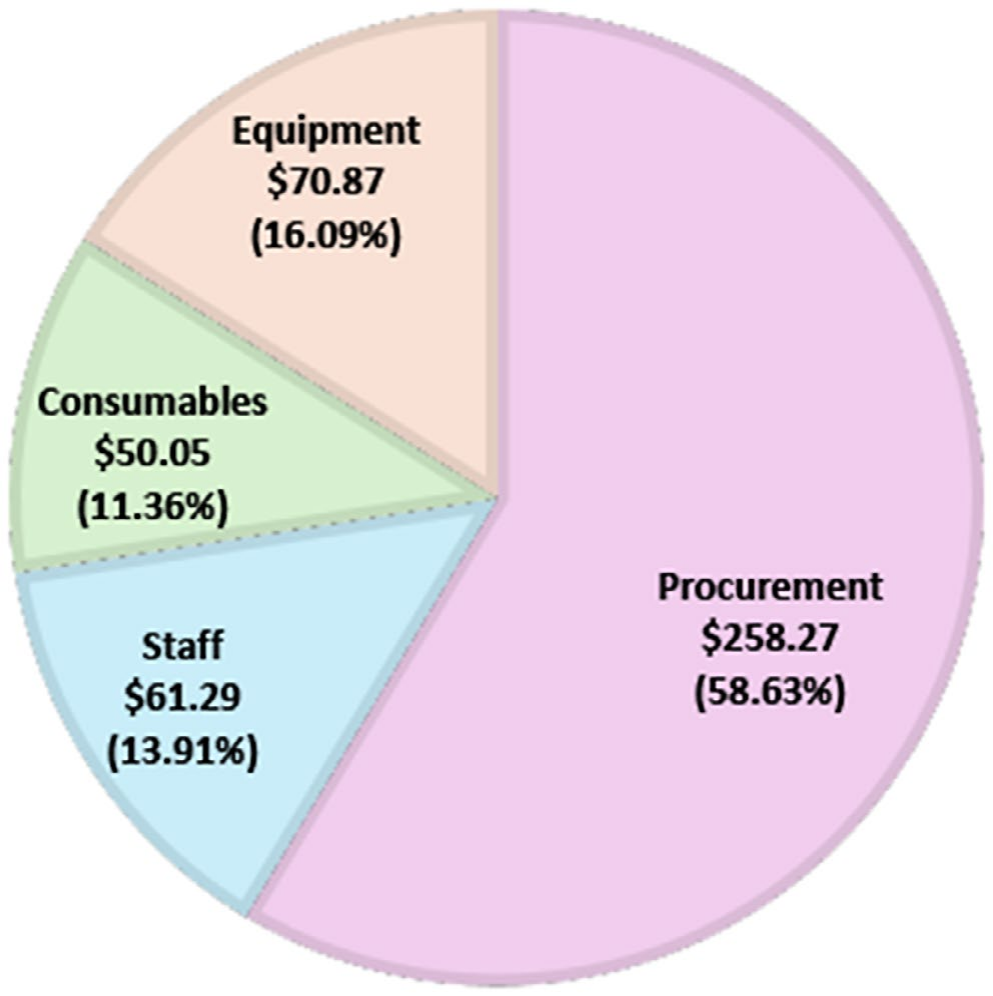
Proportion contributions of staff, equipment and consumables to overall cost of one RBC unit (US$). These proportions include the contributions of staff, consumables and equipment within each variable process and all fixed costs (including fixed overheads such as transfusion governance oversight). RBCs, red blood cells.

Of the overall cost, the biggest contributor was the product procurement price (US$258.27, 58.63%) (Figure 3; Table 3). Fixed costs contributed 24.10% (total US$106.14). These included activities required for each RBC unit, including costs of routine laboratory quality control and quality assurance activities, oversight and governance activities and equipment required in both the laboratory and the wards to enable transfusion to occur. Variable process costs contributed 17.27% (total US$76.06) (Table 3).

**TABLE 3:** Cost per RBC unit.

Cost component	Cost per unit (US$)
Procurement price of RBC unit	258.27
Variable process costs	76.06
1a, 1b Automated group and screen	8.49
2a, 2b, 33a, 33b Day prior to transfusion: OP1 & OP2 nursing/HMO duties	4.03
3a, 3b, 4a, 4b Cross‐match	2.07
5, 8a, 8b, 11 Antibody identification	0.23
7a, 7b, 7c, 7d, 7e Reference laboratory send away (genotyping, complex antibody identification, donor group discrepancy)	<0.01
9, 10 Pathology and transfusion laboratory specimen reception process	1.26
12a, 12b, 12c Transfusion reaction investigation, analysis, notification	7.16
16a Patient phenotyping, 16b Donor phenotyping	0.05
18a, 18b, 18c Direct anti‐globulin test	0.01
21, 22a, 22b, 23, 32a Phlebotomy collection	11.68
24, 25 & 28 Red cell administration process	35.07
26 Elution	1.87
27c Pharmacy dispensing iron chelation	0.15
29b Blood group discrepancy investigation	<0.01
30a, 30b, 30c Transport of RBC unit (from transfusion lab to wards and patient)	3.61
31 Transfusion laboratory issue of RBC unit	0.36
Fixed costs per RBC unit^a^	106.14
6 Donor group checks	0.08
15a1, 15a2, 15a3, 15a4, 15b1, 15c1, 15c2, 16b1 Transfusion lab QC: blood grouping, polyspecific anti‐globulin test, anti‐IgG anti‐globulin test, Rh/K phenotyping, IAT, ABD reverse & confirmation	0.59
15d, 15e1 Staff participation in internal and external QA	0.80
15f, 15g, 15h, 15i Analyser QC and maintenance	2.28
17a, 17b, 27a, 27b Daily inventory checks and RBC ordering	0.23
19a Fixed cost OP1 Daily including pharmacy	0.81
20a, 20b, 20c, 20d 20e, 20f, 20g Fridge checks & maintenance	1.69
21b, 23b, 34 Outpatient pathology fixed costs including pathology collectors duties	0.19
Transfusion governance oversight	5.13
Transfusion laboratory oversight	1.87
Haematologist oversight of MDS patient transfusion	6.67
Pharmacy oversight	0.05
IP, OP1, OP2 oversight	17.39
Clinical equipment (blood pump, IV stand, vital signs monitor, thermometer)	14.63
Transfusion laboratory equipment (card rack, automatic pipette, centrifuges, cell washer, heatblock, magnifying lamp, test tube rack, analysers, waterbath, fridge)	28.84
Enrolment in quality assurance programmes	24.88
Total	440.47

*Note*: Numbers may not add up exactly due to rounding off. Costs of less than $0.01 have been noted as <0.01.

Abbreviations: CAT, column agglutination technology; HMO, House Medical Officer (junior doctor); IAT, indirect anti‐globulin test; IgG, Immunoglobuin G; IP, inpatient ward; MDS, myelodysplastic syndromes; OP1, outpatient ward 1; OP2, outpatient ward 2; QA, quality assurance; QC, quality control; RBCs, red blood cells.

^a^ Fixed costs include fixed processes (indicated by the accompanying flowchart reference number, fixed equipment costs necessary for each RBC unit transfused, other fixed costs such as transfusion oversight or quality assurance programmes).

After analysis of individual contributions of staff salaries, equipment costs and consumables within both fixed and variable costs, total equipment costs were US$70.87 (16.09%), total staff salaries were US$61.29 (13.91%) and total consumable costs were US$50.05 (11.36%) (Figure 3).

### Yearly cost of transfusion

During the 6‐month study period, a patient with MDS received a median of 7.5 RBC units with a median of 1 RBC unit transfused per episode (Table 1), giving a total estimated yearly cost of RBC transfusion per patient of US$6607.12.

### Sensitivity analyses

#### Effect of timings variation

Although each step was timed a minimum of three times to reduce variability, there may still be variations in the timings which can impact costs. Thus, to assess the effect of a variation in timings, a sensitivity analysis was performed using ±10% of all recorded timings, resulting in a cost ranging from US$437.86 to US$443.09.

#### Effect of probabilities variation

As probabilities were either calculated using representative data from the study period (and hence may be subject to variation) or based on expert opinion, a ±10% variation on the probabilities of individual steps occurring was performed. This sensitivity analysis resulted in a cost ranging from US$423.43 to US$448.06.

### Transfusion in patients with antibodies

**FIGURE 4:**
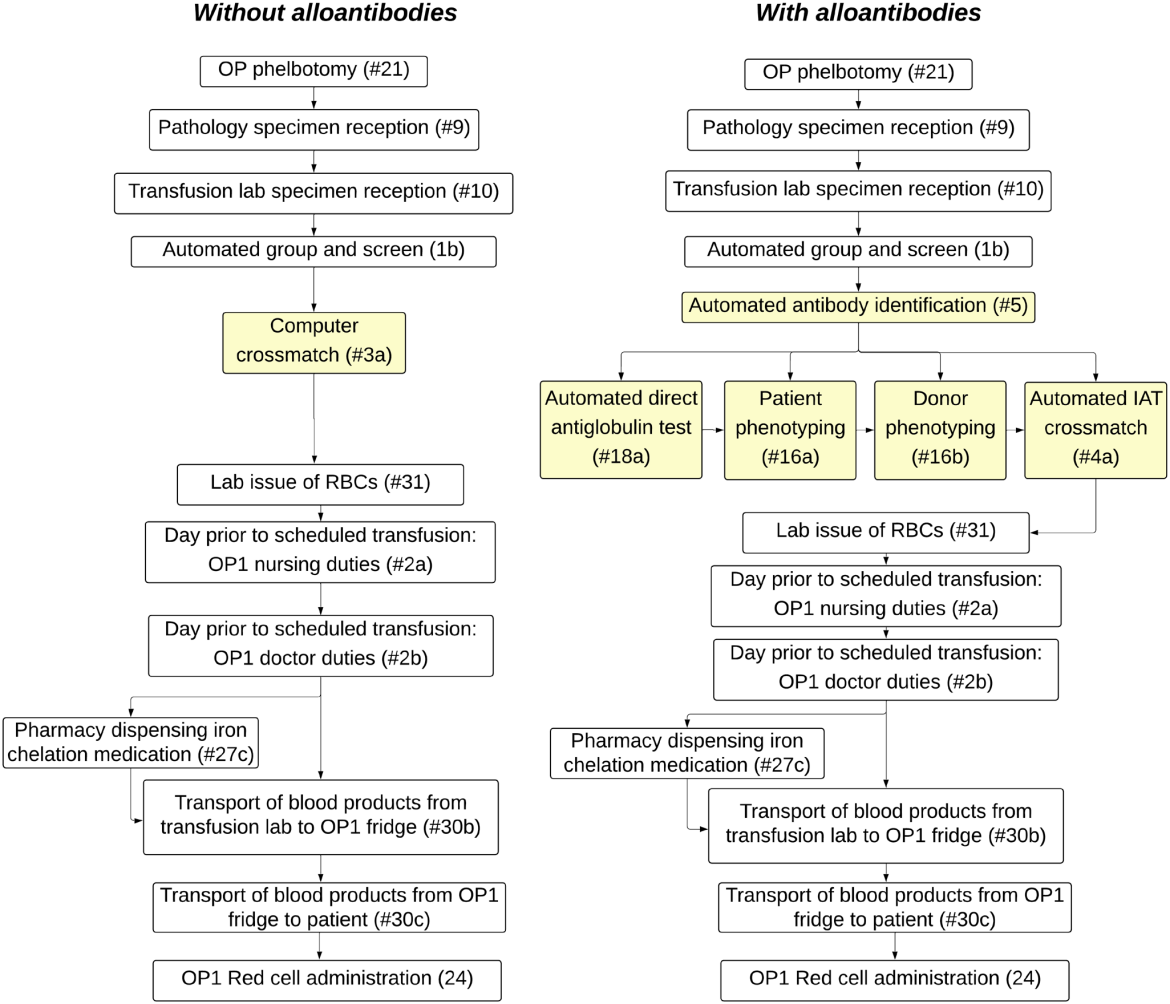
Comparing transfusion process for outpatient according to presence of alloantibodies. Flowcharts used in the costing of a scenario comparing transfusion of an outpatient in outpatient ward 1 without versus with alloantibodies. The main differences are highlighted in yellow. IV, intravenous; OP, outpatient; OP1, outpatient ward 1; RBCs, red blood cell.

Alloimmunisation results in increased complexity of transfusion processes. To calculate potential increased costs of providing RBC units to alloimmunised patients, two different scenarios were costed (Figure 4). For an outpatient transfused in outpatient ward 1, the cost of the transfusion process for one RBC unit (excluding the product procurement price and fixed costs) was US$88.47 for a patient without alloantibodies versus US$109.90 for a patient with a simple antibody (such as Rh or K antibodies) indicating a 24.22% increase in process costs due to alloimmunisation.

## DISCUSSION

In this prospective observational study, we found the total cost of providing one RBC unit to a patient with MDS is US$440.47, and the yearly cost of RBC transfusion is US$6607.12. The main contributor was the RBC product cost (58.63%). Of hospital‐related costs, the largest contributor was equipment (16.09%) followed by staffing (13.91%). Alloimmunisation increased process costs by 24%.

These findings are important as patients with MDS frequently require RBC transfusion and the prevalence of MDS is expected to rise in coming decades, due to an ageing population and population growth.^27^ Accurate costing information for transfusion support is essential for policymakers and the community to properly plan for the likely increase in RBC transfusion demand and to enable cost‐effectiveness analysis of emerging treatments for MDS‐related anaemia.

Alloimmunisation is common in patients with MDS,^10^ affecting more than 10% of patients in an Australian registry study.^28^ We simulated the processes and costs required for a patient with a simple antibody such as Rh or K, which are the most commonly detected antibodies in patients with MDS.^28^ We found that RBC transfusion process costs increased by 24% due to alloimmunisation. It is likely that costs will be even higher for patients with multiple alloantibodies, which can occur in nearly half of alloimmunised MDS patients.^28^ This has implications for transfusion laboratory staffing and resources.

Previous studies evaluating the economic burden of MDS and transfusion are largely retrospective. A systematic review of the annual costs of MDS management, including non‐transfusion treatments, found that more than 90% of included studies were either database analyses or retrospective studies, with widely ranging results from US$6777 to US$521 141.^29^ Another retrospective study estimated an average total cost of transfusion (including RBC and platelet products) ranging from US$1335 for very low‐risk revised international prognostic scoring system (IPSS‐R) MDS to US$22 560 for very high‐risk IPSS‐R MDS^30^; however, this was based upon estimates of labour and laboratory costs, and estimation of transfusion administration times rather than using an ABC approach.

TDABC approaches using direct observation of all processes have been used in previous transfusion studies. We conducted a similar TDABC study in Australian patients with thalassaemia and found a much higher RBC unit cost of US$695.59. In the thalassaemia study, the main contributing cost was iron chelation (more than 40% of the total cost and 80% of process costs).^7^ In comparison, iron chelation was rarely used in our MDS patient cohort, likely due to shorter anticipated overall survival, comorbidities and concerns regarding potential toxicity in older patients. Closer comparison of other shared processes between the two studies showed similarity in the process maps and costs of individual laboratory and clinical processes, indicating that these may be broadly applicable to a wider patient cohort beyond MDS and thalassaemia.

Other international ABC studies, while not specific to patients with MDS, have largely reported similar results to our study. A UK micro‐costing study, with a similar approach to measure all transfusion‐related activities, found that the total cost of the RBC was 40% higher than the cost of the product itself; although that study was conducted in a different country, we also found similarly that the RBC unit cost contributed approximately 59% of the total cost.^31^ A French ABC study found the process costs to be 138.41 euros (approximately 41%) of the total average cost of 339.64 euros per RBC unit transfused, though the timings for each step were evaluated by a practice survey rather than direct observation.^32^ An older US costing study from 1998 of all haematology outpatients (not just MDS) found higher proportions of fixed and variable process costs (which contributed approximately 85% of the total cost for transfusion)^33^; however, that study used interviews to estimate timings rather than direct observation and, given the data are nearly 30 years old, hospital processes have likely changed since then.

There were some limitations to our study. We did not include societal or patient‐incurred costs (e.g. lost productivity, travel expenses); while we acknowledge these are highly important to patients and families, the analysis was conducted from a healthcare system perspective. Hospital overhead costs (e.g. buildings, cost of utilities), along with broader hospital and laboratory processes not directly related to transfusion in patients with MDS, were outside the scope of the analysis; therefore, our result may be an underestimate. Government procurement prices were assumed to capture blood collection and processing, although these likely omit societal and donor‐incurred costs. This study was conducted at a single centre. Given the context‐specific nature of TDABC studies, which rely on detailed observation and data collection, similar process mapping and micro‐costing would need to be undertaken at other sites to accurately characterise variations across settings. We partially addressed this through sensitivity analyses by varying all probabilities and time parameters to reflect different contexts. Our findings are broadly consistent with other international studies using similar TDABC and/or micro‐costing approaches.^31^, ^32^ Finally, severe transfusion reactions were not observed due to their rarity; hospital data and expert consensus were used to estimate their cost, which had minimal impact on the total cost.

Strengths of our study include its detailed design and conduct in observing each step, however, simple or complex, of the entire transfusion process within the hospital. These real‐world observations increase the accuracy and relevance of our calculations. Our data also have broader implications and potential use: for example, given the similarities with previous studies in Australia and internationally, our contemporary calculated costs and process maps may be applicable to transfusion in other clinical settings. The process maps may also be examined for opportunities for improvement in transfusion efficiency and safety.

## CONCLUSION

Using a time‐driven activity costing approach, we have calculated the true cost of transfusing a unit of RBCs in patients with MDS from a healthcare provider perspective. Process costs within the hospital contributed approximately two‐fifths of the cost, and alloimmunisation increases this further. Our data may be used in future cost evaluation studies of therapies for MDS‐related anaemia, and to help hospitals and government project future costs of supplying transfusion. Future research is needed to also encompass the societal costs of transfusion to both patients and blood donors.

## AUTHOR CONTRIBUTIONS

**Kylie Rushford:** Investigation; writing – review and editing. **Angelene Jesurajah:** Investigation; writing – review and editing. **Jessica Guglielmino:** Investigation; writing – review and editing. **Philomina Banahene:** Investigation; writing – review and editing. **Dan Andrew:** Investigation; writing – review and editing. **Amanda Ellison:** Investigation; writing – review and editing. **Erin Hu:** Investigation; writing – review and editing. **Allison Mo:** Conceptualization; investigation; writing – original draft; methodology; writing – review and editing; formal analysis; project administration; data curation. **Jake Shortt:** Conceptualization; writing – review and editing; supervision; methodology. **Alayna Carrandi:** Formal analysis; writing – review and editing. **Helen Haysom:** Conceptualization; investigation; writing – review and editing; methodology; project administration; formal analysis; data curation. **Alisa M. Higgins:** Conceptualization; formal analysis; writing – review and editing; methodology. **Sara Carrillo de Albornoz:** Writing – review and editing; investigation. **Loo Sin Hoo:** Investigation; writing – review and editing. **Erica M. Wood:** Conceptualization; writing – review and editing; supervision; methodology. **Zoe K. McQuilten:** Conceptualization; writing – review and editing; supervision; methodology.

## FUNDING INFORMATION

The authors acknowledge the financial support of the Blood Synergy program, established under the Australian National Health and Medical Research Council's Synergy Grants (1189490). AM receives PhD scholarship funding from the National Health and Medical Research Council (NHMRC: APP1151512), National Blood Authority, Monash University and Haematology Society of Australia and New Zealand. AMH, JS and ZKM are supported by Australian NHMRC Emerging Leadership Fellowships (grant no. ZKM: 1194811, JS: 2009177, AMH: 2008447). EMW is supported by an NHMRC Leadership Fellowship (grant no. 1177784). Monash Health acknowledges the support of a donation from Dr. Alexander Baxter for its MDS research program.

## CONFLICT OF INTEREST STATEMENT

JS has served on advisory boards for Bristol Myers Squibb, Otsuka, Mundipharma and Novartis. JS has received research funding from Astex Pharmaceuticals. JS has received speaker's fees from Mundipharma and Novartis (all disclosures for JS are outside of the submitted work). EMW and ZKM have received research funding to their institution from: Abbvie, Amgen, Antengene, AstraZeneca, Beigene, Bristol‐Myers Squibb, CSL Behring, Gilead, GSK, Janssen‐Cilag, Novartis, Pfizer, Roche, Sanofi and Takeda and research support from Sobi, all outside the submitted work. AMH is a Director of Empiric Health. All other authors do not have any conflict of interest to disclose for the submitted work.

## ETHICS STATEMENT

This study was approved by the Monash Health Research Ethics Committee (reference number RES‐22‐0000‐007Q‐81488) and study procedures were in accordance with the ethical standards of the Research Ethics committee and with the Helsinki Declaration of 1975 (revised 2013).

## PATIENT CONSENT STATEMENT

As per ethics committee review, individual patient consent was not required as no identifiable patient information was collected.

## Supporting information


**Figure S1.** Examples of process maps.

**Table S1.** Decision diamond probabilities.

**Table S2.** Weighted cost of each process.

**Table S3.** Consumables, equipment and staff included in cost calculations.

## Data Availability

The data that support the findings of this study are available from Monash Health. Restrictions apply to the availability of these data, which were used under license for this study. Data are available from the author(s) with the permission of Monash Health.
