# Supplementary material for: The true cost of red cell transfusion for patients with myelodysplastic syndromes: A time‐driven activity‐based costing study
Source: Br J Haematol. 2026 May 21;209(1):275–85. doi: 10.1111/bjh.70556 (PMC13340471; doi:10.1111/bjh.70556)

Supplementary figure 1: Examples of process maps

Abbreviations for process maps

BGAB Blood Group and Antibody screen
CAT Column Agglutination Technology
DOB Date of Birth
EMR Electronic Medical Records
G&S Group and screen
IV Intravenous
IAT Indirect Antiglobulin Test
Lab Laboratory
LIS Laboratory information system
MDS Myelodysplastic syndromes
OP1Outpatient ward 1
OP2 Outpatient ward 2
PPE Personal Protective Equipment
RBC Red blood cell
RN Registered nurse
Tx Transfusion
XM Crossmatch

Please note that only one process map example from each phase of the transfusion process has been included in this supplementary material. The remaining process maps are available at request from the corresponding author.

Example of a phlebotomy process map

Flow chart 22A: Phelobtomy pathology collection for inpatients

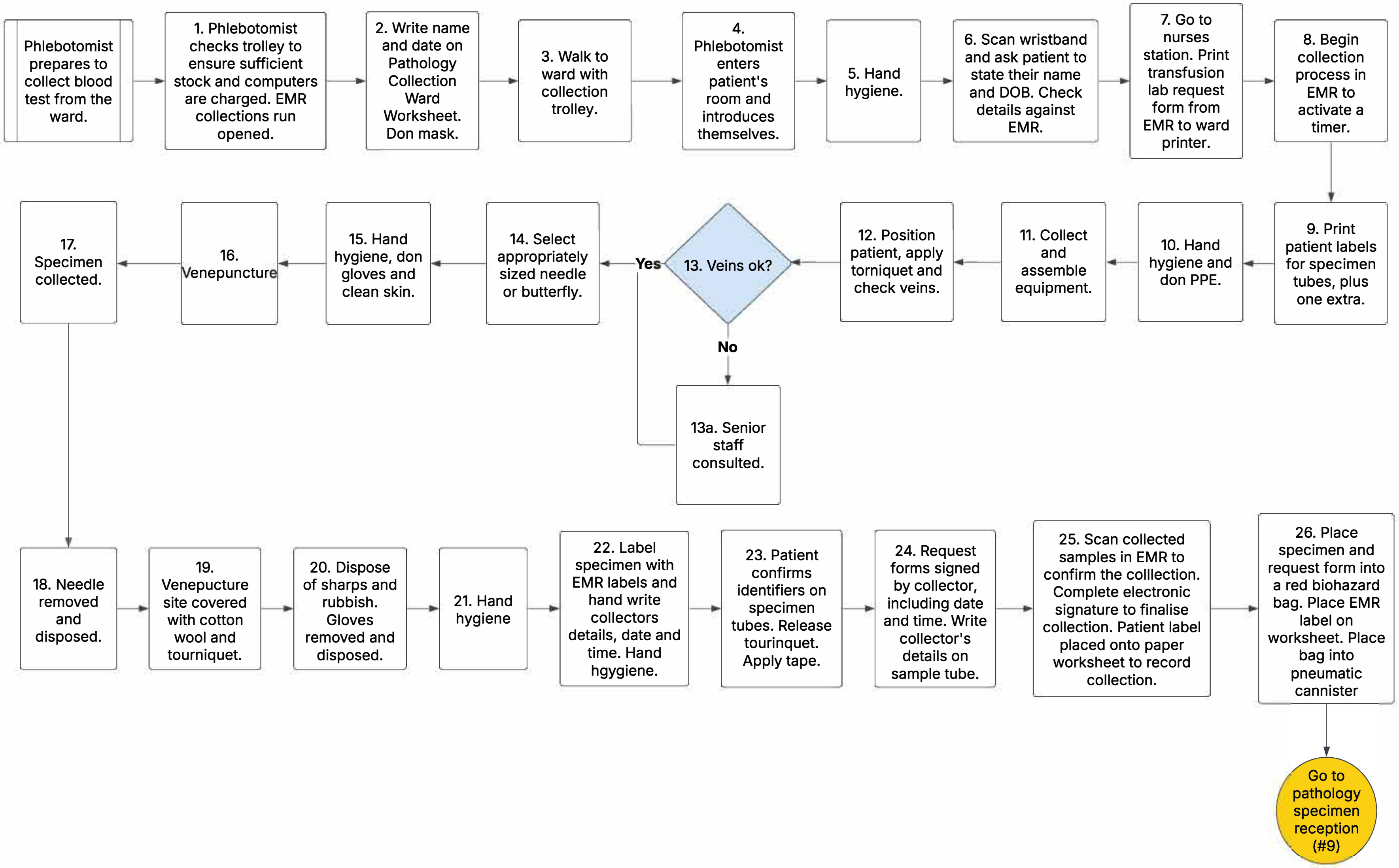

Example of a specimen reception process map

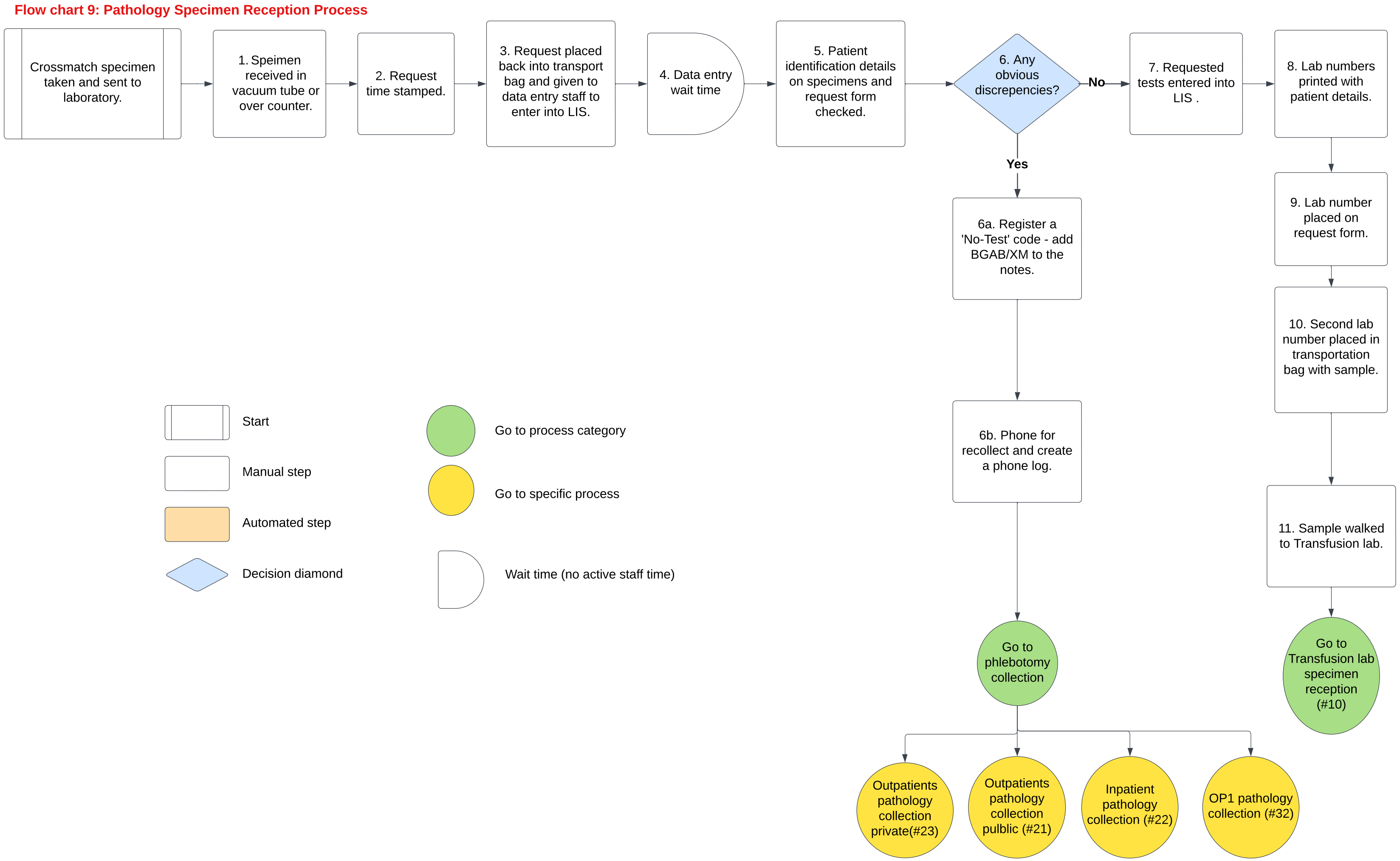

Example of a transfusion laboratory process map

Chart 1A: Automated Group and Screen Process (new patient)

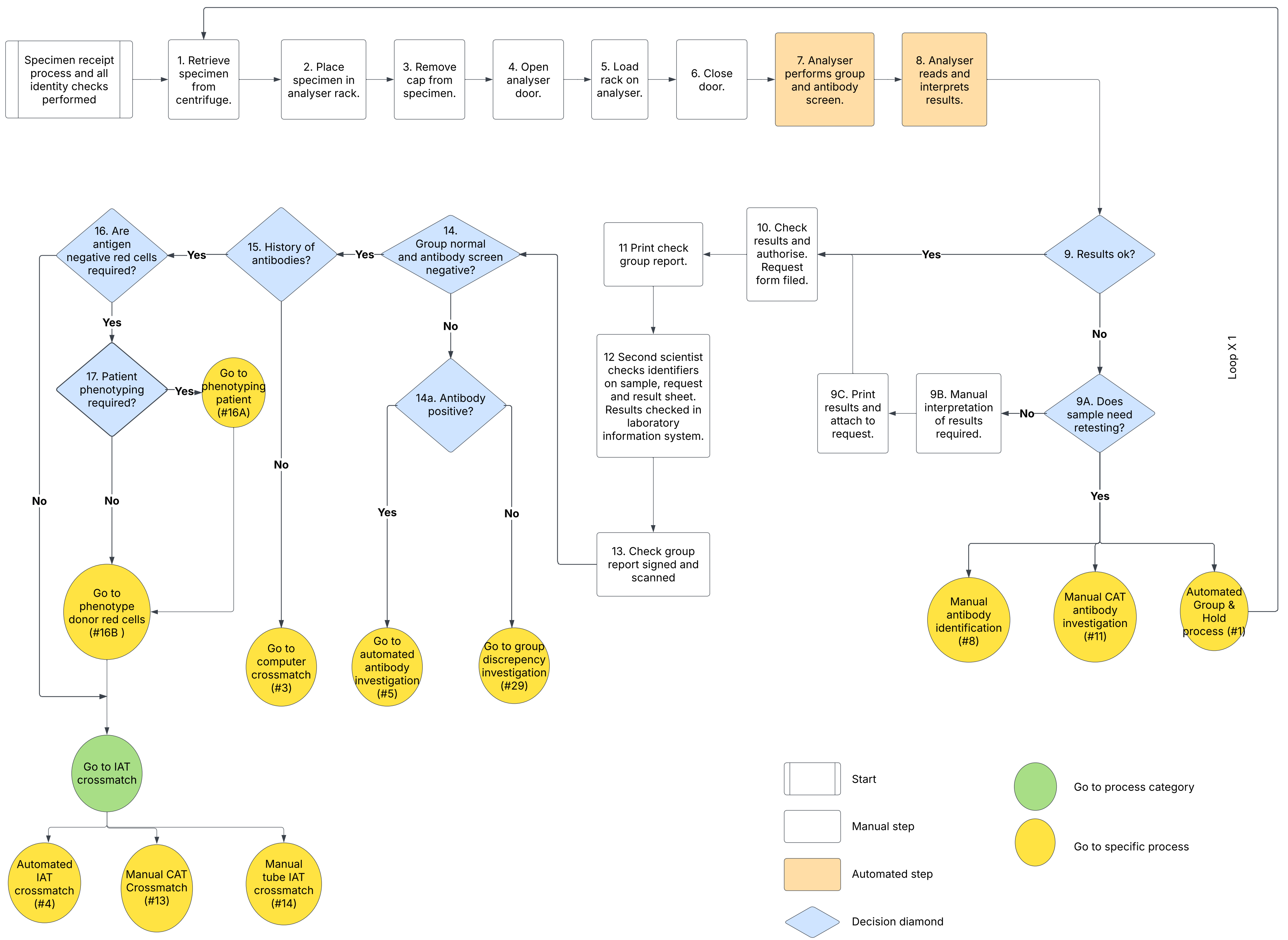

Example of a laboratory issue of blood products process map

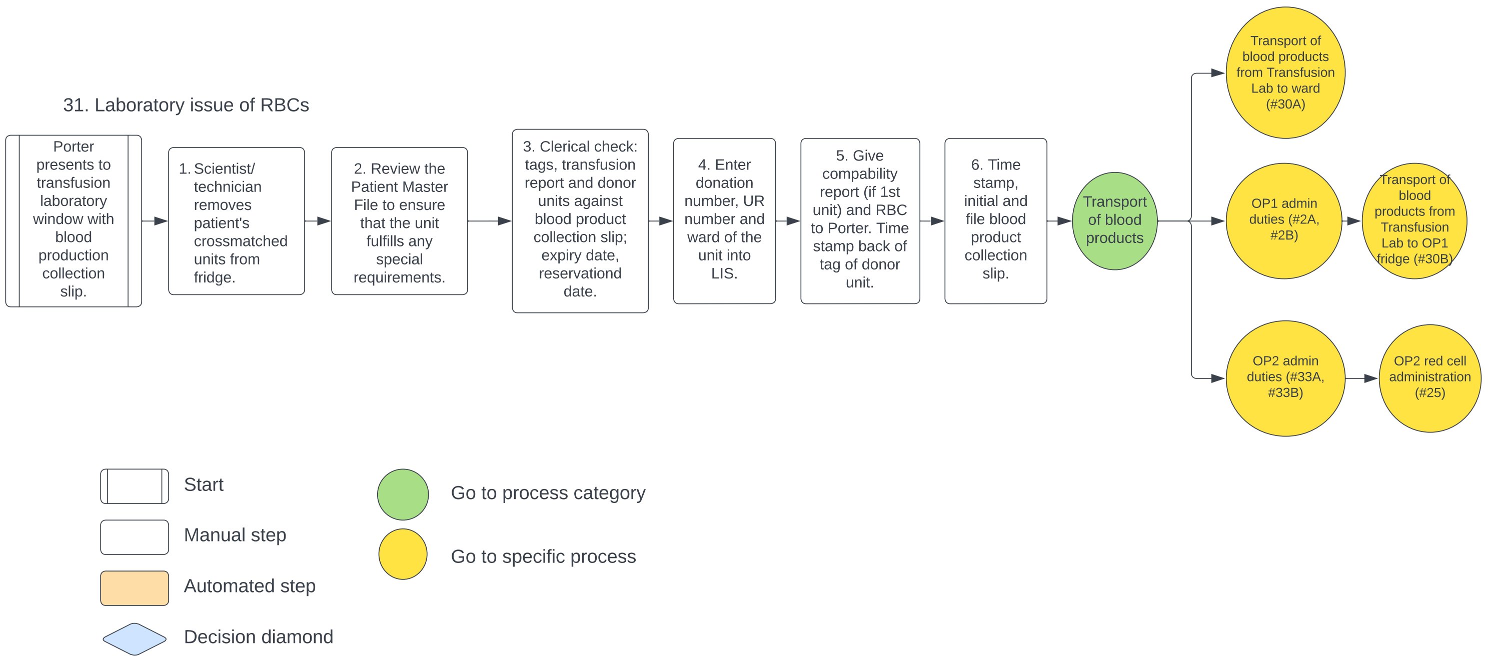

Chart 28: Ward Transfusion Process

Example of a clinical transfusion process map

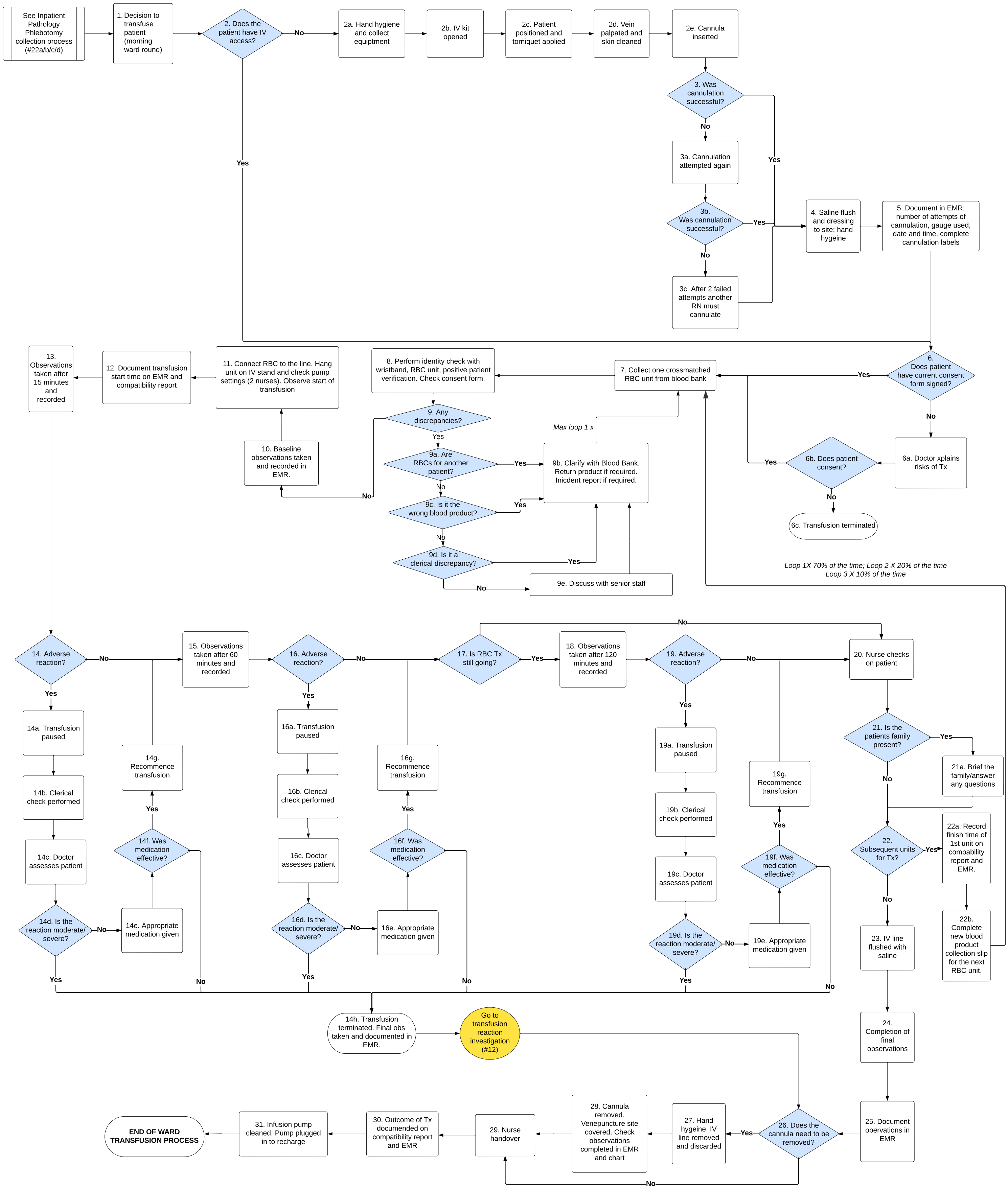

Supplement: Supplementary file 1 — Figure S1. Examples of process maps. [file BJH-209-275-s003.pdf]
