# Supplementary material for: The true cost of red cell transfusion for patients with myelodysplastic syndromes: A time‐driven activity‐based costing study
Source: Br J Haematol. 2026 May 21;209(1):275–85. doi: 10.1111/bjh.70556 (PMC13340471; doi:10.1111/bjh.70556)
Supplement: Supplementary file 2 — Table S1. Decision diamond probabilities. [file BJH-209-275-s002.pdf]

Supplementary table 1: Decision diamond probabilities

Flow Chart	Step Number	Decision diamond question	Yes probability	No probability	Source
1a and 1b		Note that flow chart 1a will occur for 5% of patients (new patients), and flow chart 1b for 95% of patients (known patients)			
1a. Automated group and screen (new patient) - 5% of the time	9	Results ok?	90%	10%	Expert opinion
	9A	Does sample need retesting?	20%	80%	Expert opinion
	Yellow circle	If yes: 49.5% manual tube, 1% manual CAT, 49.5% automated			Expert opinion
	14	Group normal and antibody screen negative?	97.70%	2.30%	Representative Data
	14a	Antibody positive?	2.30%	97.70%	Representative Data
	15	History of antibodies?	12.40%	87.60%	Representative Data
	16	Are antigen negative red cells required?	90%	10%	Expert opinion
	17	Patient phenotyping required?	0.5%	99.5%	Representative Data
	Green circle	Go to IAT Crossmatch: Automated 100%; Manual CAT 0%; Manual tube 0%	see column c	see column c	Representative Data
1b. Automated group and screen (new patient) - 95% of the time	9	Results ok?	90%	10%	Expert opinion
	9A	Does sample need retesting?	20%	80%	Expert opinion
	Yellow circle	If yes: 49.5% manual tube, 1% manual CAT, 49.5% automated			Expert opinion
	11	Group concordant and antibody screen negative?	97.70%	2.30%	Representative Data
	11a.	Antibody positive?	2.30%	97.70%	Representative Data
	12	History of antibodies?	12.40%	87.60%	Representative Data
	13	Are antigen negative red cells required?	90%	10%	Expert opinion
	Green circle	Go to IAT Crossmatch: Automated 100%; Manual CAT 0%; Manual tube 0%	see column c	see column c	Representative Data
2. OP1 admin Duties	2A Day prior to scheduled transfusion: nursing duties				
	3	Are both results available?	60%	40%	Expert opinion
	3B	Pathology tests completed externally?	5%	95%	Expert opinion

	Green circle	Go to phlebotomy collection: outpatients public 39% outpatients private 12% OP1 collection 49%	see column c	see column c	Representative Data
	2B HMO duties				
	5	Prescription required?	10%	90%	Expert opinion
3a and 3b		3a happens 95% of the time, 3b happens 5% of the time	see column c	see column c	Expert opinion
3a. Computer crossmatch with paper request form (95% of the time)	2	Has the XM request been added to the existing G&S?	70%	30%	Expert opinion
	3	Valid sample?	99%	1%	Expert opinion
	Green circle	Go to phlebotomy collection: 11% OP public, 3% OP private, 11% OP1, 75% IP	see column c	see column c	Representative Data
	4	Computer crossmatch?	99%	1%	Representative Data
		If step 4 is No: IAT Crossmatch: Automated 100%; Manual CAT 0%; Manual tube 0%	see column c	see column c	Representative Data
3b. Computer crossmatch with EMR request (5% of the time)	2	Valid sample?	99%	1%	Expert opinion
	Green circle	Go to phlebotomy collection: 11% OP public, 3% OP private, 11% OP1, 75% IP	see column c	see column c	Representative Data
	3	Computer crossmatch?	99%	1%	Representative Data
		If step 3 is No: IAT Crossmatch: Automated 100%; Manual CAT 0%; Manual tube 0%	see column c	see column c	Representative Data
4. Automated IAT crossmatch 4a. Paper request (95% of the time)	16	Results ok?	99%	1%	Expert opinion
	16a.	Does crossmatch need automated retesting?	100%	0%	Representative Data
	Green circle	Go to manual IAT crossmatch methods: % manual tube IAT XM vs Manual CAT IAT XM	100%	0%	Representative Data
	17	Units compatible?	90%	10%	Expert opinion
	17a.	Antibody investigation required?	1%	99%	Expert opinion
	16	Results ok?	99%	1%	Expert opinion

4. Automated IAT crossmatch 4b. EMR request (5% of the time)	16a.	Does crossmatch need automated retesting?	100%	0%	Representative Data
	Green circle	Go to manual IAT crossmatch methods: % manual tube IAT crossmatch vs Manual CAT IAT crossmatch	100%	0%	Representative Data
	17	Units compatible?	90%	10%	Expert opinion
	17a.	Antibody investigation required?	1%	99%	Expert opinion
5. Automated antibody identification	7	Results ok?	95%	5%	Expert opinion
	7a.	Does sample need retesting?	95%	5%	Expert opinion
	10	Is the autocontrol positive?	6%	94%	Representative data
	10a	Is an elution required?	0.50%	99.50%	Representative data
	11	Further testing/exclusions required?	90%	10%	Expert opinion
	12	New patient or new antibody?	5%	95%	Expert opinion
	14	Are antigen negative red cells required?	90%	10%	Expert opinion
	15	Patient phenotyping required?	0.5%	99.5%	Expert opinion
	Green circle	Go to antibody identification: Manual tube 50%; manual CAT 0%; automated 50%			
	Green circle	Go to IAT Crossmatch: Automated 100%; Manual CAT 0%; Manual tube 0%	see column c	see column c	Representative Data
6. Donor group checks	17	Blood group results agree?	99.99%	0.01%	Expert opinion
	17a	Is this the first restest?	99%	1%	Expert opinion
	17e.	Sendaway to ARCLB required?	1%	99%	Expert opinion
7. ARCLB Send away process	7a. ARCLB - Request for Genotyping				
	2	Is there sufficient patient sample to send-away?	95%	5%	Expert opinion
	Green circle	Go to phlebotomy collection: 11% OP public, 3% OP private, 11% OP1, 75% IP			Representative Data
	7b. ARCLB - Request for Antibody Identification				
	2	Is there sufficient patient sample to send-away?	90%	10%	Expert opinion
	Green circle	Go to phlebotomy collection: 11% OP public, 3% OP private, 11% OP1, 75% IP			Representative Data
	7d. Reviewing ARCLB results				

	6	History of antibodies?	20.00%	80.00%	Expert opinion
	7	Are antigen negative red cells required?	90%	10%	Expert opinion
	8	Patient phenotyping required?	0.50%	99.50%	Expert opinion
	Green circle	IAT crossmatch: 100% automated IAT XM vs 0% manual CAT IAT vs 0% manual IAT crossmatch	see column c	see column c	Expert opinion
	7e. ARCLB: Donor group check discrepancy				
	5	ARCLB notification: OK to add unit back to inventory?	10%	90%	Expert opinion
8. Manual antibody identification	8. Manual antibody identification				
	Chart 8a vs 8b	Probability of doing 8a 95%; probability of doing 8b is 5%	see column c	see column c	Expert opinion
	8a. Manual tube LISS IAT antibody identification: for exclusions				
	21	Autocontrol strongly positive?	10%	90%	Expert opinion
	21a	Is elution required?	5%	95%	Expert opinion
	22	Is negative control positive?	5%	95%	Expert opinion
	23	Further testing/exclusions needed?	10%	90%	Expert opinion
	24	New patient or new antibody?	5%	95%	Expert opinion
	24c	Specialist testing required?	5%	95%	Expert opinion
	24d	Does patient require phenotyping /genotyping?	90%	10%	Expert opinion
	Yes after 24d	Go to ARCLB send away genotyping: 85%; Go to patient phenotyping : 15%	see column c	see column c	Expert opinion
	26	Antigen negative red cells required?	90%	10%	Expert opinion
	Green circle	IAT crossmatch: 100% automated IAT XM vs 0% manual CAT IAT vs 0% manual IAT crossmatch	see column c	see column c	Expert opinion
	8b. Manual tube LISS IAT antibody identification: full panel				

	21	Autocontrol strongly positive?	10%	90%	Expert opinion
	21a	Is elution required?	5%	95%	Expert opinion
	22	Is neg control positive?	5%	95%	Expert opinion
	23	Further testing/exclusions needed?	10%	90%	Expert opinion
	24	New patient or new antibody?	5%	95%	Expert opinion
	24c	Specialist testing required?	5%	95%	Expert opinion
	24d	Does patient require phenotyping/ genotyping?	90%	10%	Expert opinion
	Yes after 24d	Go to ARCLB send away genotyping: 85%; Go to patient phenotyping : 15%	see column c	see column c	Expert opinion
	26	Antigen negative red cells required?	90%	10%	Expert opinion
	Green circle	IAT crossmatch: 100% automated IAT XM vs 0% manual CAT IAT vs 0% manual IAT crossmatch	see column c	see column c	Expert opinion
9. Pathology specimen reception	6	Any obvious discrepancies?	1%	99%	Representative Data
	Green circle	Go to phlebotomy collection: 3% OP private; 11% OP public, 75% IP; 11% OP1	see column c	see column c	Representative data
10. Specimen reception blood bank	2	Sample accepted by specimen reception?	100%	0%	Representative Data
	8	Any discrepancies?	1%	99%	Representative Data
	Green circle	Go to phlebotomy collection: 3% OP private; 11% OP public, 75% IP; 11% OP1	see column c	see column c	Representative data
11. Manual CAT antibody investigation	13	Is the autocontrol positive?	6%	94%	Representative data
	13a	Is an elution required?	0.50%	99.50%	Representative data
	14	Further testing/exclusions required?	90%	10%	Expert opinion
	15	New patient or new antibody?	5%	95%	Expert opinion
	16	Does patient require phenotyping /genotyping?	90%	10%	Expert opinion
	Yes after 16	Go to ARCLB send away genotyping: 85%; Go to patient phenotyping : 15%	see column c	see column c	Expert opinion
	17	Antigen negative red cells required?	90%	10%	Expert opinion

	Green circle	Go to antibody identification: Manual tube 50%; manual CAT 0%; automated 50%			
	Green circle	Go to IAT Crossmatch: Automated 100%; Manual CAT 0%; Manual tube 0%	see column c	see column c	Representative Data
12. Transfusion reaction investigation	12A.Request for Transfusion Reaction Investigation & Post-Tx sample				
	2	Appropriate documentation/sample received?	70%	30%	Expert opinion
	4	All clerical checks correct?	99.95%	0.05%	Expert opinion
	7	Have units been returned?	90%	10%	Expert opinion
	7b.	Has RBC unit been discarded/unavailable?	10%	90%	Expert opinion
	12B. Pre-Transfusion Sample				
	3	All clerical checks correct?	99.95%	0.05%	Expert opinion
	12C. Automated Testing				
	13	Do all results confirm initial testing?	99%	1%	Expert opinion
	14	Crossmatch compatible?	95%	5%	Expert opinion
	15	Elution required?	20%	80%	Expert opinion
	16	Does donor unit phenotyping need to be performed?	10%	90%	Expert opinion
	12D. Analysis and Notification				
	4	Online notification form complete?	50%	50%	Expert opinion
	7	Is transfusion reaction serious?	15%	85%	Expert opinion
13. Manual CAT IAT XM	21	Units compatible?	90%	10%	Expert opinion
14. Manaul Tube IAT XM	21	Units compatible?	90%	10%	Expert opinion
15. Reagent Quality Control	no decisions				
15G. Analyser maintenance	4	Is QC ok?	70%	30%	Expert opinion
	6	Is QC ok?	80%	20%	Expert opinion

	8	Is QC ok?	90%	10%	Expert opinion
	10	Is QC ok?	80%	20%	Expert opinion
16. Phenotyping	16A Patient Phenotyping				
	1	Can phenotyping be done?	15%	85%	Expert opinion
	2	Other monoclonal antibody typing required?	2.30%	97.70%	Representative data
	3	Other polyclonal antibody typing required?	0	100%	Representative data
	4	Will polyclonal typing be performed manually?	5%	95%	Expert opinion
	Green circle	Go to crossmatch: 100% automated IAT XM vs 0% manual CAT IAT vs 0% manual IAT crossmatch	see column c	see column c	Expert opinion
	16B Donor Phenotyping				
	1	Rh/K typing required?	2.80%	97.20%	Representative data
	2	Other monoclonal antibody typing required?	2.30%	97.70%	Representative data
	3	Other polyclonal antibody typing required?	0	100%	Representative data
	4	Will polyclonal typing be performed manually?	5%	95%	Expert opinion
	Green circle	Go to crossmatch: 100% automated IAT XM vs 0% manual CAT IAT vs 0% manual IAT crossmatch	see column c	see column c	Expert opinion
17. Inventory checks and ordering	no decisions				
18 Direct Antiglobulin Test (DAT)	18A vs 18B vs 18C	Probability of doing 18A is 99%; probability of doing 18B 0.2%, probability of doing 18C 0.8%			
	18A. Automated DAT				
	4	Is DAT strongly positive?	30%	70%	Expert opinion
	4c	Are antigen negative red cells required?	90%	10%	Expert opinion
	6	Results OK?	95%	5%	Expert opinion

	6a	Does sample need retesting?	20%	80%	Expert opinion
	Green circle	Go to original process: 5. automated 50%, Manual antibody 50%; manual CAT 0%			
	18B. Manual CAT DAT				
	5	Is DAT positive?	30%	70%	Expert opinion
	Green circle	Go to original process: 5. automated 50%, Manual antibody 50%; manual CAT 0%			
	18C. Manual tube DAT				
	5	Is DAT positive?	30%	70%	Expert opinion
	Green circle	Go to original process: 5. automated 50%, Manual antibody 50%; manual CAT 0%			
20. Blood fridge checks and maintenance	20A Daily (blood bank) Fridge Check				
	4	Are there any discrepancies?	1%	99%	Expert opinion
	20B Daily (OP1) Fridge Check (Mon-Sat)				
	5	Are there any discrepancies?	1%	99%	Expert opinion
21. Outpatient pathology public	16	Veins OK?	85%	15%	Expert opinion
22. Inpatient phlebotomy		Probability of 22A is 91%; probability of 22B is 9%	see column c	see column c	Expert opinion
	22a. Pathology collection via venepuncture by phlebotomy staff				
	13	Veins OK?	60%	40%	Expert opinion
	27 .	Are there more patients requiring ward pathology collections?	93%	7%	Expert opinion

	22b. Pathology collection by ward staff				
	4	Is there an existing cannula to take bloods?	60%	40%	Expert opinion
	22c. Ward staff venepuncture				
	6	Veins ok?	60%	40%	Expert opinion
	21	Send specimens to pathology (% for the 3 options 21a =60%, 21b=30%, 21c = 10%)	see comment	see comment	Expert opinion
	22d. Ward staff collection via cannula				
	21	Send specimens to pathology (% for the 3 options 21a =60%, 21b=30%, 21c = 10%)	see comment	see comment	Expert opinion
23. Outpatient pathology phlebotomy private	16	Veins OK?	85%	15%	Expert opinion
24. OP1 Red cell administration	1	Has patient arrived?	99%	1%	Expert opinion
	1b	Is patient attending?	50%	50%	Expert opinion
	8	Is patient well?	90%	10%	Expert opinion
	8b	Will patient be transfused?	95%	5%	Expert opinion
	9	Does the patient have current consent form signed?	80%	20%	Expert opinion
	9b	Does patient consent?	99%	1%	Expert opinion
	14	Was cannulation successful?	90%	10%	Expert opinion
	14b	Was cannulation successful?	99%	1%	Expert opinion
	17	Are crossmatched RBC available?	95%	5%	Expert opinion
	17a	Does patient have valid group and screen?	95%	5%	Expert opinion
	18	Are patient units in the blood fridge?	50%	50%	Expert opinion

	21	Any discrepancies?	0.01%	99.99%	Expert opinion
	21 options	21a RBCs for another patient 0.5%. 21b Wrong blood product 0.5%. 21c Clerical discrepancy 99% 21d Other discrepancy 0%	see column c		Expert opinion
	26	Adverse reaction?	0%	100%	Representative Data
	26d	Is the reaction moderate/severe?	5%	95%	Expert opinion
	26f	Was medication effective?	50%	50%	Expert opinion
	28	Adverse reaction?	0%	100%	Representative Data
	28d	Is the reaction moderate/severe?	5%	95%	Expert opinion
	28f	Was medication effective?	50%	50%	Expert opinion
	29	Is the RBC Tx still going?	91%	9%	Representative Data
	31	Adverse reaction?	0%	100%	Representative Data
	31d	Is the reaction moderate/severe?	5%	95%	Expert opinion
	31f.	Was medication effective?	50%	50%	Expert opinion
	32	Does patient have transport home organised?	80%	20%	Expert opinion
	38	Subsequent units for transfusion?	18.35%	81.65%	Representative data
25.OP2 Red cell administration	5	Was pre-transfusion testing done at hospital pathology?	89%	11%	Representative Data
	8	Has haematology resident responded within 20 mins?	50%	50%	Expert opinion
	14	Does patient have current consent form signed?	80%	20%	Expert opinion
	21	Was cannulation successful?	90%	10%	Expert opnion
	21b	Was cannulation successful?	90%	10%	Expert opinion
	21e	Was cannulation successful?	95%	5%	Expert opinion
	26	Sample accepted by Blood Bank?	90%	10%	Expert opinion
	27	Does patient require saline line to keep lines open?	5%	95%	Expert opinion
	30	Are Xmatched RBCs available?	20%	80%	Expert opinion

	35	Any discrepancies?	1%	99%	Expert opinion
	35a	Are RBCs for another patient?	0.01%	99.99%	Expert opinion
	35b	Is it the wrong blood product?	0.01%	99.99%	Expert opinion
	35c	Is it a clerical discrepancy?	1%	99%	Expert opinion
	40	Adverse reaction?	0%	100%	Representative Data
	40d	Is the reaction moderate/severe?	0%	0%	Representative Data
	40f	Was the medication effective?	0%	0%	Representative Data
	42	Adverse reaction?	0%	100%	Representative Data
	42d	Is the reaction moderate/severe?	0%	0%	Representative Data
	42f	Was the medication effective?	0%	0%	Representative Data
	44	Adverse reaction?	0%	100%	Representative Data
	44d	Is the reaction moderate/severe?	0%	0%	Representative Data
	44f	Was the medication effective?	0%	0%	Representative Data
	45	Is the RBC Tx still going?	77%	23%	Representative data
	47	Adverse reaction?	0%	100%	Representative data
	47d	Is the reaction moderate/severe?	0%	0%	Representative Data
	47F	Was the medication effective?	0%	0%	Representative Data
	48	Does patient need toileting assistance?	95%	5%	Expert opinion
	52	Subsequent units for transfusion?	56%	44%	Representative Data
26. Elution	1	Senior deems elution appropriate	2%	98%	Expert opinion
	13	Results ok?	90%	10%	Expert opinion
	13A	Does eluate need to be retested?	90%	10%	Expert opinion
	13B	Sufficient eluate to retest?	50%	50%	Expert opinion
	13D	Does elution need to be repeated?	5%	95%	Expert opinion
	13E	Is sample recollection required?	20%	80%	Expert opinion
	Green circle	Go to phlebotomy collection: 11% OP public, 3% OP private, 11% OP1, 75% IP	see column c	see column c	Representative Data

27. OP1 pharmacy duties.	27a. OP1 Medicines Impress				
	8	Are refrigerated medicines on the requisition?	9.50%	90.50%	Representative Data
	9	Are IV fluids on the requisition?	100%	0	Representative Data
	13	Does quantity of stock on requisition match what was taken?	99%	1%	Expert opinion
	27B. OP1 saline imprest stock				
	11	Does quantity of stock on requisition match what was taken?	99%	1%	Expert opinion
	27C. Pharmacist daily duties: dispensing iron chelation medications				
	2	Is the prescription legal, clear and valid?	99%	1%	Expert opinion
	4	Are there any changes in medication or dosage?	80%	20%	Expert opinion
	7	Is the requested drug administered subcutaneously?	0%	100%	Representative Data
	10	Are water ampules required?	0%	100%	Representative Data
	12	Any discrepancies?	1%	99%	Expert opinion
	15	Same day script?	83%	17%	Expert opinion
28. IP Ward Transfusion Process	2	Does the patient have IV access?	90%	10%	Expert opinion
	3	Was cannulation successful?	70%	30%	Expert opinion
	3b	Was cannulation successful?	90%	10%	Expert opinion
	6	Does patient have current consent form signed?	80%	20%	Expert opinion
	6b	Does patient consent?	99.50%	0.50%	Expert opinion
	9	Any discrepancies?	20%	80%	Expert opinion
	9a	Are RBCs for another patient?	60%	40%	Expert opinion

	9c	Is it the wrong blood product?	5%	95%	Expert opinion
	9d	Is it a clerical discrepancy?	35%	65%	Expert opinion
	14	Adverse reaction?	4%	96%	Representative Data
	14d	Is the reaction moderate/severe?	25%	75%	Representative Data
	14f	Was medication effective?	80%	20%	Representative Data
	16	Adverse reaction?	5%	95%	Representative Data
	16d	Is the reaction moderate/severe?	5%	95%	Representative Data
	16f	Was medication effective?	100%	0%	Representative Data
	17	Is RBC Tx still ongoing?	89%	11%	Representative Data
	19	Adverse reaction?	0%	100%	Representative Data
	19d	Is the reaction moderate/severe?	0%	0%	Representative Data
	19f	Was medication effective?	0%	0%	Representative Data
	21	Is the patient's family present?	50%	50%	Expert opinion
	22	Subsequent units for Tx?	11%	89%	Expert opinion
	26	Does the cannula need to be removed?	99%	1%	Expert opinion
29. Group discrepancy investigation	29a. Group Discrepancy Investigation overview				
	2	Has the patient been recently transfused with a different blood group?	90%	10%	Expert opinion
	2a	Can the result be adequately explained by another reason?	90%	10%	Expert opinion
	4	Is senior scientist review necessary?	5%	95%	Expert opinion
	4b	Specialist testing required?	80%	20%	Expert opinion
	Green circle	Go to crossmatch process: Computer crossmatch 99%; IAT crossmatch 1%	see column c	see column c	Representative Data
	Green circle	Go to IAT Crossmatch: Automated 100%; Manual CAT 0%; Manual tube 0%	see column c	see column c	Lab Representative Data
	29b. Blood group discrepancy investigation				

	11	Incubation required?	95%	5%	Expert opinion
	12	Forward and reverse group discrepant and extra testing required?	10%	90%	Expert opinion
	13	What further testing required? 13a. Repeat testing 10%; Go to ARCLB sendaway 40%; 13b Incubate at different temp 50%	see column c	see column c	Expert opinion
	Green circle	Go to crossmatch 99%; manual tube LISS IAT antibody full panel 0%; automated antibody identification 1%	see column c	see column c	Representative data and expert opinion
	Green circle	Go to crossmatch: computer crossmatch 99%; automated crossmatch 1%; manual CAT 0%; manual tube 0%	see column c	see column c	Representative data and expert opinion
	Probabilities of 30A vs 30B	The probability depends on whether the transfusion is in the ward or in OP1. For ward transfusions, the probability of 30A is 100% and 30B is 0%. For OP1 transfusions, the probability of 30A is 50% and 30B is 50% (based on expert ANUM opinion). 30C only happens if 30B happens.	see column c	see column c	Expert opinion
30 Transport of blood products	30B Transport of blood products from blood bank to OP1 fridge				
	7	Was > 1 unit collected?	62%	38%	Representative Data
	30 C Transport of blood products from OP1 fridge to OP1 patient				

		2. Is the blood unit in the fridge?	50%	50%	Expert opinion
31 Lab issue RBCs	Green circle	Probability of transporting to: ward 69%; transport to OP1 fridge 25%; going to OP2 RBC transfusion 6%			
32 OP1 Pathology Collection	32a OP1 Pathology Collection Process				
	4	Is patient receiving a transfusion immediately after blood specimens are taken (urgent transfusion)?	5%	95%	Expert opinion
	Yellow circle	Go to outpatients pathology: OP pathology public 76%; OP pathology private 24%	see comment	see comment	Representative Data
	32b. OP1 cannulation and blood collection				
	8	Was cannulation successful?	90%	10%	Expert opinion
	8b	Was cannulation successful?	95%	5%	Expert opinion
	20	Send sample to pathology.	99% chute, 1% PSA		Expert opinion
	33a. Week prior to scheduled transfusion: nursing duties				
	4	Is Hb results available?	70%	30%	Expert opinion
	4a	Are pathology tests external?	30%	70%	Expert opinion

33 OP2 admin duties	6	What is Hb level?	see comment	see comment	Expert opinion
	6c	Is transfusion required?	70%	30%	Expert opinion
	Green circle	Outpatients pathology public 76% Outpatients pathology private 24%	see column c	see column c	Representative Data

Abbreviations:
ARCLB Australian Redcross Lifeblood (Reference Laboratory), CAT Column Agglutination Technology, DAT Direct Antigloblin Test, EMR Electronic Medical Records, G&S Group and Screen, Hb Hemologlobin, IAT Indirect Antiglobulin Test, IP Inpatient, IP Inpatient ward, IV intravenous, OP Outpatient, OP1 Outpatient ward 1, OP2 Outpatient ward 2, QC Quality Control, RBC Red Blood Cells, Path pathology, Tx Transfusion, XM Crossmatch
