# Supplementary material for: The true cost of red cell transfusion for patients with myelodysplastic syndromes: A time‐driven activity‐based costing study
Source: Br J Haematol. 2026 May 21;209(1):275–85. doi: 10.1111/bjh.70556 (PMC13340471; doi:10.1111/bjh.70556)
Supplement: Supplementary file 3 — Table S2. Weighted cost of each process. [file BJH-209-275-s004.pdf]

Supplementary table 2: Weighted cost of each process

Process	Total cost of process (USD)
Variable processes	
1a Automated group and screen (new patient)	8.50
1b Automated group and screen (known patient)	8.49
2a Day prior to scheduled transfusion: nursing duties in OP1	13.60
2b Day prior to scheduled transfusion: HMO duties in OP1	0.97
3a Computer crossmatch with paper request form	0.58
3b Computer crossmatch with electronic medical records request	0.60
4a Automated IAT crossmatch with paper request	10.46
4b Automated IAT crossmatch with electronic medical records request	10.67
5 Automated antibody identification	30.28
7a Reference laboratory: request for genotyping	1.32
7b Reference laboratory: request for antibody identification	2.03
7c Specimen reception send-away process	1.29
7d Reviewing reference laboratory results	0.80
7e Reference laboratory: donor group check discrepancy	3.93
8a Manual tube IAT antibody identification for exclusions	3.87
8b Manual tube IAT antibody identification full	10.26
9 Pathology specimen reception process	0.27
10 Transfusion laboratory specimen reception	0.96

11 Manual CAT antibody investigation	11.26
12a Request for transfusion reaction investigation and post-transfusion sample	1.27
12b Pre transfusion sample testing (transfusion reaction)	0.35
12c Automated testing (transfusion reaction)	28.50
12d Analysis and notification (transfusion reaction)	36.59
16a Patient phenotyping	1.17
16b Donor phenotyping	0.40
18a Automated DAT	7.86
18b Manual CAT DAT	8.27
18c Manual tube DAT	1.55
21 Outpatient pathology phlebotomy (public)	9.29
22a Pathology collection via venepuncture by phlebotomy staff	12.23
22b Pathology collection by IP ward staff	8.35
23 Outpatient pathology phlebotomy (private)	9.28
24 OP1 red cell administration	53.52
25 OP2 red cell administration	61.74
26 Elution	92.13
27c Pharmacist daily duties: dispensing iron chelation medications	5.92
28 IP ward red cell administration	26.08
29b Blood group discrepancy investigation	0.18
30a Transport of blood products from transfusion laboratory to ward	0.83

30b Transport of blood products from transfusion laboratory to OP1 fridge	1.91
30c Transport of blood products from OP1 fridge to OP1 patient	6.84
31 Laboratory issues of RBCs	0.36
32a OP1 pathology collection process	0.44
32b OP1 cannulation and blood collection	26.81
33a Week prior to scheduled transfusion: nursing duties in OP2	5.53
33b Week prior to scheduled transfusion: doctor duties in OP2	1.03
Fixed processes	
6 Donor group checks	3.67
15a1 Blood group QC (daily)	2.55
15a2 Antiglobulin test polyspecific QC (daily)	1.72
15a3 Antiglobulin tests anti IgG QC (daily, weekdays)	2.20
15a4 Rh/Kell phenotyping QC (daily)	12.13
15b Manual CAT IAT QC (weekly)	9.29
15c1 ABDReverse, ABD confirmation and poly AHG cassettes QC (monthly)	23.20
15c2 Polyspecific AHG (monthly)	2.52
15d1 AIMS Quality Assurance Program participation by every staff member, annually	70.27
15d2 RCPA Quality Assurance Program (general) participation by every staff member, annually	66.68

15d3 RCPA Quality Assurance Program (phenotyping) participation by every staff member, annually	59.08
15e1 Manual tube internal replicate testing, participation by every staff member, twice a year	17.99
15e2 Manual CAT internal replicate testing, participation by every staff member, twice a year	30.72
15f Analyser daily probe maintenance	4.78
15g Analyser daily QC, three times a day	35.04
15h Analyser weekly maintenance	1.94
15i Analyser monthly maintenance	2.31
17a Daily inventory checks and RBC ordering (general stock)	4.05
17b Daily inventory checks and RBC ordering (special orders for MDS patients)	0.33
19a Fixed cost OP1 daily	0.53
20a Daily transfusion laboratory fridge check	0.31
20b Daily OP1 remote fridge check	1.03
20c Weekly OP1 remote fridge check	1.27
20d Monthly fridge maintenance transfusion laboratory	1.99
20e Monthly remote fridge maintenance OP1	0.84
20f Transfusion laboratory 6 monthly checks and maintenance	7.12
20g Transfusion laboratory annual checks and maintenance	9.49
21b OP pathology fixed costs (public)	0.40
23b OP pathology fixed costs	0.40

27a OP1 pharmacy duties	3.32
27b Pharmacy costs	3.12
34 Pathology collectors’ daily duties	3.41

Table 3 legend:

Abbreviations: AIMS: Australian Institute of Medical and Clinical Scientists, CAT Column Agglutination Technology, DAT Direct Antiglobulin Test, HMO Hospital Medical Officer (junior doctor), IAT Indirect Antiglobulin Test, IP Inpatient ward, MDS Myelodysplastic syndromes, OP1 Outpatient ward 1, OP2 Outpatient ward 2, QC Quality Control, RCPA Royal College of Pathologists of Australasia
