# Supplementary material for: The true cost of red cell transfusion for patients with myelodysplastic syndromes: A time‐driven activity‐based costing study
Source: Br J Haematol. 2026 May 21;209(1):275–85. doi: 10.1111/bjh.70556 (PMC13340471; doi:10.1111/bjh.70556)
Supplement: Supplementary file 4 — Table S3. Consumables, equipment and staff included in cost calculations. [file BJH-209-275-s001.pdf]

Supplementary table 3: consumables, equipment and staff included in cost calculations

CONSUMABLES
0.8% A1 and B cells, 10mL
A1 cells
B cells
B reverse grouping cells
0.9% normal saline 10ml ampoule
0.9% saline (lab use)
0.9% normal saline 100ml bag
0.9% normal saline 50mL bag
Water ampoules
Low ionic strength saline
Sodium hydroxide
10 ml syringes
3mL EDTA tube
10ml EDTA tube
22 guage needle
24 guage needle
7% bovine serum albumin
A4 paper
ABD confirmation card
ABD reverse cassette
Antibody screen cell 1
Antibody screen cell 2
Antibody screen cell 3
Anti human globulin (AHG) control cells
AHG poly card
Anti-A antisera
Anti-AB antisera
Anti-B antisera
Anti-c antisera
Anti-C3d antisera
Anti-C antisera
Anti-D antisera
Anti-E antisera
Anti-e antisera
Anti-Fya antisera
Anti-Fyb antisera
Anti-Jka antisera
Anti-Jkb antisera
Anti-k antisera
Anti-Kpa antisera
Anti-lea antisera
Anti-leb antisera
Anti-m antisera

Anti-n antisera
Anti-p1 antisera
Anti-s antisera
Anti-S antisera
Anti-IgG AHG
PolyAHG reagent
PolyAHG casette
Red cell reagent 3%
Red cell reagent 0.8%
Monoclonal antisera
Antisera donor group check
Antibody register card
Antigen composition sheet
Group and reverse cards
Group check card
Compatability report
Container for diluted wash solution
RAM reagent used for pre transfusion testing
Elution kits
Direct antigobulin test card
Monospecific direct antiglobulin test card
Neutral column agglutination technology (CAT) card
Dilution tray (16 wells)
Thermoplastic film
Pipette tips
Plastic bag
Plastic tile
Plastic pocket
Plastic transfer pipettes
Worksheets for lab
Donor identification number labels
Evaporation caps
Glass test tubes
Protective glasses
Disposable non-sterile gloves
Segmenting safety device
Tags for blood product
Labels for blood products
Alcohol swab
Alcohol sanitiser
Alcohol wipes
Antiseptic spray
Patient appointment card
Band-aid dot
Intravenous pump infusion blood set
Rubbish bin liner

Biohazard bag
Biohazard bin liner
Blood products consent form
Blood bank collection slip
Blood bank request form
Urine specimen pot
Patient label
Butterfly pathology collection set
Cannula
IV infusion clamp
Cotton ball
Dressing
Gauze
Handwash
Patient wristband
Infusion chart
IV cannula insertion pack
IV kit
Medication chart
N95 mask
Needle
Paracetamol
Furosemide
Hydrocortisone
Adrenaline
Loratidine
Iron chelator medication
Patient parking voucher
Pathology request form
Rubber bands
Sterile gloves
Tape
Tourniquet
Transfusion reaction form
Transparent dressing
Vacutainer barrel
Thermometer probe cover
EQUIPMENT
Automatic pipette
Centrifuge
Heatblock
Blood bank fridge
Bloodbank record book
Blood pump
Card rack for laboratory testing
Cellwasher

Magnifying lamp
Phenotyping rack
Reusable safety classes
Satellite fridge to store blood products, in ward
Test tube rack
Vein finder
Blood bank analyser
Volumetric pipette
Waterbath
IV stand
Cost of enrolment in quality assurance programs X 3
Thermometer
Vital signs monitor
STAFF
Ward administration officer
Haematology consultant
Junior doctor
Laboratory assistant
Medical scientist
Pathology collector
Pathology team leader
Pharmacist
Pharmacy technician
Porter
Registered nurse
Transfusion nurse specialist
Senior scientist
Specimen reception staff
Nurse unit manager / senior nurse
Staff involved in transfusion governance (senior clinicians involved in transfusion committee meetings)
